# Cancer-related cognitive problems at work: experiences of survivors and professionals

**DOI:** 10.1007/s11764-019-00830-5

**Published:** 2019-11-25

**Authors:** Kete M. Klaver, Saskia F. A. Duijts, Ellen G. Engelhardt, Chantal A. V. Geusgens, Maureen J. B. Aarts, Rudolf W. H. M. Ponds, Allard J. van der Beek, Sanne B. Schagen

**Affiliations:** 1 Division of Psychosocial Research and Epidemiology, Netherlands Cancer Institute, Plesmanlaan 121, 1066 CX Amsterdam, The Netherlands; 2 Department of Public and Occupational Health, Amsterdam Public Health Research Institute, Amsterdam UMC, Vrije Universiteit Amsterdam, Amsterdam, The Netherlands; 3 Netherlands Comprehensive Cancer Organisation (IKNL), Utrecht, The Netherlands; 4 Division of Molecular Pathology, Netherlands Cancer Institute, Amsterdam, The Netherlands; 5 Department of Clinical and Medical Psychology, Zuyderland MC, Sittard, The Netherlands; 6 Department of Medical Oncology, Maastricht University MC, Maastricht, The Netherlands; 7 Department of Medical Psychology/School of Mental Health and Neurosciences (MHeNS), Maastricht University MC, Maastricht, The Netherlands; 8 Brain and Cognition Group, University of Amsterdam, Amsterdam, The Netherlands

**Keywords:** Focus group, Cancer, Work, Cognitive problems, Employability

## Abstract

**Purpose:**

Cancer-related cognitive problems (cancer-related cognitive problems) in working cancer survivors are found to affect work outcomes. We aimed to generate in-depth information regarding cancer-related cognitive problems in working cancer survivors, strategies used to cope with cancer-related cognitive problems at work, and needs of cancer survivors and professionals regarding cancer-related cognitive problems at work.

**Methods:**

Five focus groups were formed, amongst which three focus groups with cancer survivors (*n* = 8, *n* = 7, and *n* = 8) and two focus groups with professionals (*n* = 7, *n* = 8). Thematic analysis of the transcripts was performed to create concepts.

**Results:**

Both cancer survivors and professionals confirmed that cancer-related cognitive problems, which occurred in several domains of neurocognitive functioning, affect work functioning. Cancer survivors used several strategies (e.g., applying practical adjustments, re-organization of work, and accepting limitations) to cope with cancer-related cognitive problems at work, as did professionals in their attempt at supporting cancer survivors facing these problems. Various needs of cancer survivors (e.g., supportive care options, acknowledgment by others) and professionals (e.g., improvement of expertise, clarity about referral pathways) regarding cancer-related cognitive problems at work were mentioned.

**Conclusions:**

Due to the growing number of working cancer survivors dealing with cancer-related cognitive problems, it is essential to sustain their employability. Therefore, cognitive rehabilitation interventions should be developed, taking functioning at work into account. Knowledge amongst professionals regarding cancer-related cognitive problems, as well as coordination of care for cancer-related cognitive problems, should be improved. Ensuring professional education regarding cancer-related cognitive problems, within both the healthcare and occupational setting, is of utmost importance.

**Implications for cancer survivors:**

Support for working cancer survivors who experience cancer-related cognitive problems might increase their employability in the longer term.

**Electronic supplementary material:**

The online version of this article (10.1007/s11764-019-00830-5) contains supplementary material, which is available to authorized users.

## Introduction

In the past decades, cancer survival rates have increased due to screening and improved treatment modalities. On average, 89% of cancer survivors are able to (partly) return to work (RTW) within 24 months following a cancer diagnosis [1, 2]. From cancer survivors’ perspective, paid work is associated with having a purpose in life, it contributes to psychological well-being and to have an overall sense of normalcy [2, 3]. From a societal perspective, the loss of work capacity in cancer survivors should be avoided. However, adverse work outcomes are common in cancer survivors, such as an increased risk of sickness absenteeism and of a decline in productivity levels while at work (i.e., presenteeism) and/or ability to work (i.e., an individual’s psychological, physical, and social means to engage in work [3]), compared to non-cancer groups. Moreover, cancer survivors also more frequently experience job loss and higher levels of receiving work disability benefits than the general working population [1, 3, 4]. Consequently, supporting cancer survivors to continue their work, beyond initial RTW, is increasingly important.

While it can be expected that patients diagnosed with and treated for tumors in the central nervous system (CNS) are confronted with cancer-related cognitive problems [5, 6], patients with non-CNS tumors can be confronted with these problems as well. Cancer-related cognitive problems in patients with non-CNS tumors often improve after completion of therapy and once other disease- and treatment side-effects diminish. However, a substantial subgroup (up to 35%) of cancer survivors with non-CNS tumors also exhibits persistent cancer-related cognitive problems that can last for years following completion of cancer therapy [7–9]. The potential adverse effects of cancer-related cognitive problems on functioning at work have received considerable attention. Several work-related outcomes, such as work performance and self-perceived work ability, may be affected by cancer-related cognitive problems [10–14]. For example, Dorland et al. (2017) have found work functioning, in the year following RTW, to be associated with subjective cognitive functioning. That is, cancer survivors with persistently low work functioning reported more cognitive symptoms than cancer survivors with moderate to high or persistently high work functioning [15]. Due to the potentially lasting nature of cancer-related cognitive problems, there may be a long-term impact of cancer-related cognitive problems on work outcomes [8]. For example, in cancer survivors who are able to RTW, work-related cancer-related cognitive problems appear to be stable, but continuously present, during the first 18 months post-RTW [16].

Given the increasing number of cancer survivors that are able to RTW, there is an urgent need to improve their sustained employability. Despite the body of evidence that suggests an impact of cancer-related cognitive problems on work performance and work ability [10, 11], up to now, the strategies that cancer survivors use to cope with cancer-related cognitive problems at work are not well studied. Furthermore, there remains a paucity in comprehension about the needs of cancer survivors who are dealing with cancer-related cognitive problems at work. This information is important in order to avoid adverse work outcomes in cancer survivors, such as long-term sickness absence or even work disability, due to cancer-related cognitive problems. As returning to work and continuation of work can be considered as complex processes, in which many stakeholders are involved, it is important to consider several perspectives (e.g., those from the cancer survivors, employers, and (occupational) health care professionals (HCPs)), in order to achieve sustained employability in cancer survivors.

To generate the information needed and future interventions to help cancer survivors with non-CNS tumors who experience cancer-related cognitive problems to maintain their employability, we aimed to identify (1) cancer-related cognitive problems in cancer survivors who returned to work, (2) strategies that cancer survivors use to cope with cancer-related cognitive problems at work, and strategies that professionals use in their attempt at supporting these cancer survivors, and (3) needs of cancer survivors and professionals regarding cancer-related cognitive problems at work. We used a qualitative, in-depth approach to explore the perspectives of different stakeholders (i.e., cancer survivors and professionals).

## Methods

### Design, participants selection, and recruitment

We formed focus groups with cancer survivors (i.e., people who are living with or beyond their cancer. Thus, people who have completed treatment or who are having ongoing treatment for their cancer) and separate focus groups with employer representatives (e.g., human resource consultants) and (occupational) HCP (now: professionals). Cancer survivors were eligible for participation in a focus group if they: (1) experienced cancer-related cognitive problems at work (assessed by KK during a phone call); (2) were diagnosed with a non-CNS tumor; (3) were currently employed in paid work; and (4) were able to communicate in Dutch. An invitation to participate was placed on the website of the Dutch Federation of Cancer Patient Organizations and was announced in the patient panel of the Netherlands Cancer Institute (i.e., a group of cancer patients who may be approached to discuss issues related to cancer care). Professionals were eligible for participation in a focus group if they had experience with cancer survivors dealing with cancer-related cognitive problems at work. Professionals were identified through the network of the research team and invited by phone or email. Recruitment of participants stopped when data saturation was reached.

### Procedure and data collection

In total, five focus groups were formed; three with cancer survivors and two with professionals. The focus groups were semi-structured, with a guided discussion about the following topics: cancer-related cognitive problems at work, strategies used by cancer survivors to cope and by professionals to support cancer survivors with cancer-related cognitive problems at work, and cancer survivors’ and professionals’ needs to deal with cancer-related cognitive problems at work. A list of focus group questions can be found in the supplementary table. Before the start of a focus group, all participants gave informed consent and completed a brief online questionnaire, in case of the cancer survivors about socio-demographics (e.g., age, gender, education), their work situation (e.g., working hours, type of job), cancer diagnosis (e.g., tumor type, time since diagnosis), and treatment received (e.g., modality, time since completion), and in case of the professionals about socio-demographics (e.g., age, gender) and their work situation (e.g., type of job, years of experience in their current profession). The leading moderators (SD, SS, and SZ) are researchers (i.e., clinical neuropsychologists, senior researchers) with extensive experience guiding group discussions. There was an observer (KK) present during each focus group session, who took notes and audiotaped the sessions. At the start of each focus group, the lead moderator discussed the objective and structure of the focus group. The focus groups lasted about 2 h each and took place at the Netherlands Cancer Institute in Amsterdam.

### Data analysis

The audio-recorded focus group sessions were transcribed verbatim. To safeguard the anonymity and confidentiality of participants, names were removed from the transcripts. Thematic analysis of the transcripts was performed using the standard procedure for thematic analysis described by Braun and Clarke, which consists of six phases [17]. An overview of the phases is provided in Table 1. RQDA [18], R package [19] for computer-assisted qualitative data analysis, was used to perform the thematic analyses.

**Table 1:** Phases of transcript analysis

Phase	Performed by
1. Familiarize with the data	KK, EE
2. Generate initial codes	KK, EE
3. Search for themes based on initial codes	KK, EE
4. Reviewing themes	KK, EE, SD
5. Discuss results of analysis	KK, EE, SD
6. Report results	KK, EE, SD, CG, MA, RP, SS, AB

### Focus groups coding methods

The transcript of two focus groups (i.e., one involving cancer survivors, one involving professionals) was coded independently by KK and EE. The resulting initial codebooks were then compared and refined to develop a consensus-based draft codebook. A third author (SD), not involved in the initial codebook development, was consulted to review the first consensus-based version of the codebook for cancer survivors and the codebook for professionals, prior to using it in the analysis of the remaining focus groups. In this manner, two codebooks were created, i.e., one to code the focus groups with cancer survivors and one to code the focus groups with professionals. Next, the three remaining focus groups were analyzed by one researcher (KK). A second researcher (EE) reviewed the coding and discrepancies between both were resolved until consensus was reached. The final data analysis findings were discussed with a third author (SD), not involved in the coding of the transcripts. Quotes are presented to illustrate the data. The selected quotes were translated into English by a native English speaker.

## Results

### Sample characteristics

Twenty-three cancer survivors (5 men and 18 women) with a mean age of 49.5 years (SD 11.4; range 31–70 years) participated in the focus groups (Table 2). Participants had various cancer diagnoses (e.g., breast, cervix, (colo)rectal, kidney, and melanoma), of which breast cancer was most prevalent (*n* = 6; 26%). The majority of participants (82.6%) were highly educated. The mean time since diagnosis was 61.7 months (SD 62.1; range 12–306 months). Cancer survivors worked an average of 31.5 h/week (range 12–40 h), and 61% of the cancer survivors (*n* = 14) worked full time (i.e., in the Netherlands, working ≥ 30 h a week is considered to be a full-time job), predominantly in non-manual jobs (one participant was a blue collar worker).

**Table 2:** Characteristics of focus groups participants

	Frequency (%)
Cancer survivors	*n* = 23
Sex	
Women	17 (74)
Men	6 (26)
Age at focus group, category (years)	
30–40	4 (17)
41–50	11 (48)
51–60	2 (9)
61–70	6 (26)
Education	
Low	1 (4)
Medium	3 (13)
High	19 (83)
Race/ethnicity	
White	23 (100)
Job sector^a^	
Business and financial	5 (22)
Education	4 (17)
Culture, recreation	3 (13)
Transport	4 (17)
Other^b^	15 (65)
Working hours a week	
11–20	4 (17)
21–30	6 (26)
31–40	13 (57)
Cancer diagnosis^a^	
Breast	6 (26)
Melanoma	2 (9)
Kidney	2 (9)
Cervix	4 (17)
Colorectal	2 (9)
Rectal	2 (9)
Other^c^	8 (35)
Disease stage^a^	
Localized	10 (44)
Loco-regionally advanced	13 (57)
Distant metastases	1 (4)
Treatment received^a^	
Chemotherapy	15 (65)
Immunotherapy	4 (17)
Endocrine therapy	6 (26)
Radiation	13 (57)
Surgery	19 (83)
Time since diagnosis (months)	
10–50	15 (65)
51–100	5 (22)
> 100	3 (13)
Professionals	*n* = 15
Sex	
Woman	13 (87)
Man	2 (13)
Age, category (years)	
25–30	1 (7)
31–40	4 (27)
41–50	4 (27)
51–60	5(33)
61–70	1(7)
Profession	
Human resource consultant	3 (20)
Psychologist	3 (20)
Occupational coach	2 (13)
Occupational therapist	3 (20)
Occupational physician	1 (7)
Social worker	1 (7)
Case manager in training	1 (7)
Labor expert	1 (7)
Months of experience in current profession	
0–100	7 (47)
101–200	4 (27)
201–300	3 (20)
> 300	1 (7)

^a^Multiple outcomes per person possible

^b^Industry, energy and water, construction, government, trade, health, metals and mining

^c^Ovarian, head/neck, prostate, esophagus, thyroid, leukemia, Hodgkin lymphoma, non-Hodgkin lymphoma

Fifteen professionals (i.e., human resource consultants (*n* = 3), job coaches (*n* = 2), occupational therapists (*n* = 3), occupational physician (*n* = 1), psychologists (*n* = 3), social worker (*n* = 1), case manager in training (*n* = 1), and a labor expert (*n* = 1)) participated in the focus groups. Professionals (2 men and 13 women) had a mean age of 46.8 years (SD 10.6; range 29–67) and a median of 9 years experience in their current profession.

In the following section, we will describe the results according to three themes:

1. Cancer-related cognitive problems in cancer survivors who returned to work;
2. Strategies used to manage cancer-related cognitive problems at work from a cancer survivor and professional perspective;
3. Needs of cancer survivors and professionals regarding cancer-related cognitive problems at work from a cancer survivor and professional perspective.

### Cancer-related cognitive problems in cancer survivors who returned to work

The most often reported cognitive problems at work by cancer survivors were memory problems, impaired processing speed, and attention problems. The majority of cancer survivors reported having problems with planning and executing their work. In addition, problems with fatigue and psychological distress were often mentioned by both cancer survivors and professionals in relation to cancer-related cognitive problems.

“And when I eh, worked for an hour and I didn’t finish it entirely and I came back after lunch, I had to start all over again, because I had completely forgotten what I had been working on.”

“But that’s what I find difficult when I’m working, because I often have meetings or conferences and then I just lose part of what’s going on, because my focus is gone.”

“Then it's done, then nothing gets in anymore, no. And that's right. I eh, I always say my brain these days, since I had the chemo, you know, those snowflakes? … they never seem to fall down anymore, those snowflakes.”

“Well, my impulsiveness is such that I really have to focus on doing my work. Because I’ll easily start doing something else.”

“But the slightest thing causes stress. Say, it’s decided that we need to organize an informative evening. Well, typically for me that is a piece of cake. Now, I can’t do that anymore.”

“I also had the impression, well, I'm just going to work eight hours again, but I find that around four o'clock I just breakdown and then eh, I start making mistakes or eh, things don’t register anymore.”

### Strategies used to manage cancer-related cognitive problems at work

Cancer survivors used the following strategies:

1. Putting extra effort into work: a common strategy used by cancer survivors was putting extra effort into their work to compensate for their cancer-related cognitive problems and to meet their own and others’ (e.g., supervisor and colleagues) expectations. However, concerns were expressed about this strategy, as for some cancer survivors this resulted in over-exhaustion*.*

“You are just, because you have been sick, you don’t work 100%. You work 110%. You know, you just want to be part of it all again.”

“As soon as all blood counts were good, eh, on we go, back to living a normal life. Working full time. But now I’m at home. Simply, because I was completely drained.”

2. Re-organization of work: some cancer survivors mentioned they re-organized their work (e.g., more breaks, dividing tasks into small subtasks, work less hours, switch to another (less demanding) position).

“I’ve learned to divide my work into manageable parts; read, stop, let the information sink in.”

“I take a break every one and half hours.”

3. Applying compensatory strategies: some cancer survivors reported applying compensatory strategies in order to deal with cancer-related cognitive problems at work, such as (1) taking notes, (2) working in a quiet area, (3) using feedback from others (e.g., colleagues, clients) to monitor the accuracy of their work, and (4) recording conversations.

“I am always asking my clients for feedback: Is what I´m doing still correct? Are we on the same page?”

4. Accepting limitations as a result of cancer-related cognitive problems: some cancer survivors mentioned acceptance of cancer-related cognitive problems as a strategy. A common view amongst cancer survivors is that acceptance of cancer-related cognitive problems is challenging, as it interferes with their aim to function at their “normal,” i.e., pre-cancer diagnosis, level.

“Eh, I fought so hard, eh, to come back from this, get back to normal. To work 36 hours, but well, I couldn’t. I also cried.”

5. Communicating at work about cancer-related cognitive problems: several cancer survivors mentioned that they practiced being honest and open about their cancer-related cognitive problems towards colleagues and employers as a coping strategy. However, opinions differed as to whether cancer survivors should be so forthcoming at work about their cancer-related cognitive problems. Factors influencing the decision whether or not to disclose cancer-related cognitive problems at work were acknowledgment of these problems by self and others, the working situation (e.g., risk of job loss), and the relationship with supervisor and colleagues (e.g., mutual understanding). Some cancer survivors felt that disclosure improves acknowledgment by supervisors and colleagues, while others argued that the level of understanding by others diminishes over time. Due to the persistent impact of their cancer-related cognitive problems at work, some cancer survivors were constantly forced to remind their supervisors and colleagues of their problems.

“But I keep on repeating that they really need to check me. So that’s what I say, guys when you notice weird stuff, that’s my department. I keep on harping on that, because I still make mistakes.”

6. Arranging supportive care: some cancer survivors reported that they used supportive care in order to cope with cancer-related cognitive problems at work, such as visiting a psychologist, a coach, and/or an occupational physician. Generally, cancer survivors indicated that support from professionals with expertise in cancer-related cognitive problems, fatigue, distress, and work-related issues, was helpful.

Professionals used the following strategies to support cancer survivors dealing with cancer-related cognitive problems at work:

1. Providing psycho-education: several professionals provided information about cancer-related cognitive problems and the impact of cognitive problems on daily life, as it helps cancer survivors to recognize and acknowledge these problems. A common view amongst professionals was that cancer-related cognitive problems are typically accompanied by symptoms of fatigue and psychological distress. They emphasized that clarification of symptoms is essential in order to provide adequate personalized supportive care.

“It’s important to understand the potential origins of the cognitive problems for patients, so that they realize what is happening to them.”

“First thing is to find out how it really is and what issues patients actually face . To get that all out in the open, that is a very important step. So don't go straight to the solution, but first put thought into how it is and what is going on and what it is that the patient is confronted with.”

2. Instruction to deal with fatigue: professionals mentioned that they advised strategies (e.g., monitoring fatigue during a day, taking breaks, setting boundaries) to deal with fatigue at work.

“Have your patients look at a typical day and recognize which things limit their energy.”

“I teach patients to listen to their feelings. Let them ask themselves questions like: How am I doing? What are my needs? Because many patients keep going and going, without taking any breaks.”

3. Advise on communication about cancer-related cognitive problems at work: some professionals advise cancer survivors to “be open about their cancer-related cognitive problems at work.” This is important as it leads to mutual understanding between the employee and colleagues and/or supervisor. However, professionals reported that the degree of openness about cancer-related cognitive problems varies between cancer survivors.
4. Encourage cancer survivors to take a more active role in staying at work: professionals indicated that some cancer survivors are too cautiously, and hampered their own reintegration process. Employer representatives, specifically, expressed their concern about supervising cancer survivors with a passive attitude towards reintegration, because it is difficult to keep track of their needs. Therefore, some professionals stressed the importance to encourage cancer survivors to behave pro-actively at work and to stimulate their self-esteem.

“So people who choose to wait and see, is very difficult for an employer, because then it is just a matter of searching what you can offer such a person? I think it would only be conducive, for the process of returning to work, if people themselves also get involved.”

“Sometimes people rely too much on others, so I always tell people that if they themselves have a plan, or an idea, employers and occupational physicians will be very inclined to go along with it.”

“An advantage is that people learn to regain control. And that is very important. I’m in charge again, after having to surrender to a variety of people in beautiful white coats for a long time. Control contributes tremendously to job satisfaction, to feeling strong, to feel in power.”

“And include the healthy part of people. I always ask them what their talent is. So they tell you what they are very good at.”

5. Teaching compensatory strategies: a few professionals advised cancer survivors to use compensatory strategies at the workplace (e.g., using an agenda, working in a quiet area) to deal with cancer-related cognitive problems at work.

“It is surprising how all kinds of people can benefit from some basic practical advice, for example, about organizational skills or the use of an agenda.”

### Needs of cancer survivors and professionals regarding cancer-related cognitive problems at work

The following needs of cancer survivors regarding cancer-related cognitive problems at work were mentioned:

1. Acknowledgement of cancer-related cognitive problems that can impact cancer survivors’ work: a common view amongst cancer survivors was that cancer-related cognitive problems are not well understood in our society (e.g., by (occupational) HCPs, supervisors and colleagues). Cancer survivors mentioned that the lack of acknowledgment of cancer-related cognitive problems increases psychological distress.

“Well, there are people that say, yes that chemo brain doesn’t exist, what nonsense.”

“People around you say, yes, but we are all getting older.”

2. Insight into the cognitive processes that hamper optimal functioning at work: cancer survivors reported that it is important to acquire better understanding of the cognitive processes that hamper optimal functioning at work. This might help them to anticipate their abilities and needs.

“I need clarity about my cognitive ability. I need to know what is actually possible and what is not.”

3. Tools to cope with cancer-related cognitive problems at work: some cancer survivors mentioned they are in need of tools to cope with cancer-related cognitive problems at work.

“I need practical advice to better handle cognitive problems at work, for example, during interaction with colleagues or while experiencing stress at work.”

4. Guidance to cope with fatigue: some cancer survivors reported difficulties in recognizing and establishing their own boundaries. As a result, often they feel suddenly overwhelmed by fatigue. Therefore, cancer survivors mentioned they are in need of guidance to cope with fatigue.

“At least that’s my experience, I’m at work and full of energy and then from one moment to the next it’s as if somebody flipped a switch.”

“How do I set limits? How do I recognize that I won’t finish this. Yes, what are the signs?”

“I need to figure out, when should I stop working? And when can I continue?”

5. Support for acceptance of cancer-related cognitive problems: generally, cancer survivors had difficulty accepting their cancer-related cognitive problems, and found it a time-consuming process. Some cancer survivors experienced dissonance in their perception of themselves before their cancer diagnosis and of their current self, which leads to feelings of distress (e.g., anxiety and frustration).

“In the beginning I was very angry at my brain and I thought, this is not possible and I’m very smart and I can memorize anything and I know everything, yes, apparently no longer.”

6. Support for communication about cancer-related cognitive problems at work: as some cancer survivors experienced the disclosure of their cancer-related cognitive problems at work as a burden, there is a need for advice on how to communicate this to colleagues and/or supervisors.

**“**I would like to know how to explain this to the people around me.”

“I would like to have more courage to share, because I don’t share that easily.”

7. Adequate supportive care options for cancer-related cognitive problems at work: a common view amongst cancer survivors was the difficulty of finding adequate support for cancer-related cognitive problems at work, and the lack of knowledge amongst (occupational) HCPs regarding these supportive care options. Some cancer survivors argued that physicians, nurses, and general practitioners (GPs) should provide information about cancer-related cognitive problems, and/or refer them to other specialists if necessary. However, at the moment, this rarely happens. Furthermore, cancer survivors reported different experiences with the quality of support. Overall, cancer survivors indicated that they are in need of help with finding adequate supportive care options for cancer-related cognitive impairment at work.

“It is important that referral pathways are clear.”

“I feel my specialist is very much like, eh, eh, when it is not detectable in the blood, well, then there is not much I can do. What I actually want him to say is ‘I can’t help you with this, but I can refer you to people who can.”

“Well, I think it would be nice if eh, look if you get sick and are going to be treated in a hospital, there will be a treatment plan. And cognitive problems should be part of that, as they are caused by your illness.”

“I went to see a psychologist, did EMDR, yoga, mindfulness, I did it all, on my own. And at one point I thought, yes, you have done so much, yet, the cognitive problems come back again or you still have them.”

“My occupational therapist came up with useful practical strategies to deal with cognitive problems.”

Professionals reported the following needs to be able to support cancer survivors with cancer-related cognitive problems at work:

1. Improvement of expertise regarding cancer-related cognitive problems at work: some professionals suggested that not all (occupational) HCPs are knowledgeable about the long-term effects of cancer. Therefore, it was suggested that expertise of (occupational) HCPs should be improved.

“To get in touch with a good psychologist somewhere along the way, is just a matter of luck.”

2. Clarity regarding referral pathways: some professionals argue that, due to a range of (unclear) referral options, it is difficult to obtain an overview of these options for cancer-related cognitive problems.

“Usually, there are so many HCP involved and each and every one of them has his/her own vision. That might be overwhelming for a patient.”

3. Screening: it was suggested that implementation of a screening program for long-term effects of cancer, including cancer-related cognitive problems, might facilitate professionals to offer adequate care for cancer survivors who deal with cancer-related cognitive problems at work.

“I used to work in pediatric oncology, where screening for long-term effects of cancer is standard practice. I would like such a screening for adult cancer survivors as well. Structural evaluation of how they are doing and what their needs are.”

## Discussion

### Main findings

This focus group study yields in-depth insights in experiences of cancer survivors and professionals regarding cancer-related cognitive problems at work. A common view amongst cancer survivors and professionals was that cancer-related cognitive problems occurred in several domains of neurocognitive functioning (e.g., memory, processing speed, executive functioning, and attention) and that these cancer-related cognitive problems affected work functioning. Cancer survivors used several strategies to cope with cancer-related cognitive problems at work (e.g., applying compensatory strategies, re-organization of work, accepting limitations as a result of cancer-related cognitive problems, and communicating at work about cancer-related cognitive problems), as did professionals in their attempt at supporting cancer survivors facing these problems. However, cancer survivors mentioned they experienced a lack of proper guidance. Both cancer survivors and professionals unanimously stated that knowledge amongst professionals regarding cancer-related cognitive problems, as well as coordination of care for cancer-related cognitive problems should be improved.

### Interpretation of the findings

In accordance with the present results, previous literature has demonstrated that cancer-related cognitive problems affect work functioning in non-CNS cancer survivors [10, 11, 13, 20, 21]. Previously, Von Ah et al. (2013) [22] reviewed a number of qualitative studies that evaluated strategies generally used by cancer survivors to cope with cancer-related cognitive problems. Strategies in five major areas were reported, including (1) self-management (e.g., not rushing, focusing on one task at a time), (2) physical environment (e.g., keeping things in the same place, use assistive devices), (3) social environment (e.g., open communication, seek support in social environment), (4) reducing stress and fatigue (e.g., exercise, meditation), and (5) mind-stimulating activities (e.g., Sudoku, puzzles) [22]. The current study, with its specific focus on strategies and needs of both cancer survivors and professionals in the occupational setting, showed that cancer survivors attempt to use comparable strategies at work with the addition of *compensation* (i.e., to put extra effort in work) and *acceptance*. Furthermore, both cancer survivors and professionals reported that the use of strategies at work is often unsuccessful when proper professional guidance, on how to implement such strategies, is lacking.

Both cancer survivors and professionals mentioned a shortage of (access to) interventions for cancer-related cognitive problems. Some cancer survivors, in the current study, reported that the generic supportive care they received so far was inadequate to help them cope with cancer-related cognitive problems at work, specifically. To date, several cognitive rehabilitation approaches for cancer-related cognitive problems have been developed and tested, such as cognitive strategy training, cognitive function training, physical exercise, and relaxation-based interventions. There is evidence that some of these approaches (such as cognitive strategy training) lead to improvements in self-perceived and tested cognitive function [6, 9, 23–26]. However, little is known about the specific effects of these rehabilitation interventions on work functioning. Bosma et al. (2019) suggested several elements (e.g., management of symptoms at work (i.e., worker awareness and recognition of their symptoms and limitations), finding a healthy balance, communication about limitations at work, and requesting for practical adjustments at work) as possible starting points for supportive care for workers with a chronic condition [27]. The findings of this study suggest that including those elements in cognitive rehabilitation interventions for occupational active cancer survivors might enhance their effect on work functioning. Moreover, a key barrier is that cancer-related cognitive problems may not be recognized as such in clinical practice because of the overlap between symptoms of cancer-related cognitive problems, fatigue and psychological distress [5, 8, 16]. For example, symptoms of cancer-related cognitive problems may be mistakenly interpreted as burn-out symptoms, and therefore, cancer-related cognitive problems at work are at risk of being misdiagnosed. In order for rehabilitation interventions to be effective, it is important for (occupational) HCP to be able to disentangle what symptoms cause the experienced cancer-related cognitive problems at work. Therefore, brief measures to evaluate self-reported cognitive problems, fatigue, and distress in the context of work could be of assistance in clinical practice. For example, the Cognitive Symptom Checklist–Work 21 could be used as measure for self-reported cognitive problems at work, although normative data is lacking so far [28–30].

The current study yields two key findings. First, professionals’ knowledge about cancer survivorship issues, and cancer-related cognitive problems in particular needs to be expanded. Second, there is a lack of coordination between the various professionals involved in the care for cancer survivors returning to work. Stapelfeldt et al. (2018) reported that several professionals, e.g., employers, occupational and physical therapists, and social workers, should be involved in interventions to support occupationally active cancer survivors to retain work [31]. As in the Netherlands and other Western countries, the healthcare and occupational health systems are not naturally intertwined, it is challenging to ensure structured coordination of care and greater interdisciplinary collaboration[32].

### Strengths and limitations

This study has several strengths, such as the participation of different stakeholders (both cancer survivors and professionals) and cancer survivors with a variety of cancer diagnoses, and the semi-structured, professionally guided, discussions. A limitation of this study is that nearly all cancer survivors were highly educated and worked in non-manual jobs. Consequently, the results need to be interpreted with caution and cannot automatically be transferred to cancer survivors with low educational level and those in manual jobs. Besides, this study involved some cancer survivors who did not work full time, which may have influenced their experience with cancer-related cognitive problems at work. However, since cancer survivors worked a mean time of ≥ 30 h a week and all cancer survivors worked at least 12 h a week, we believe this sample was able to give in-depth insight into the experience of cancer survivors’ cognitive difficulties at work. Furthermore, due to the variety in disciplines of professionals, each discipline was represented by a maximum of three representatives. Thus, no firm statements can be made on behalf of the separate disciplines.

### Implications and conclusion

The findings of this study have a number of important implications for (future) practice. First, cancer survivors who experience cancer-related cognitive problems in the context of work have unmet needs. Therefore, cognitive rehabilitation interventions should be developed to sustain employability of cancer survivors confronted with cancer-related cognitive problems at work. This study highlighted that approaches that cancer survivors and professionals use for some of the cognitive challenges at work, should be considered in future efforts to sustain work. For example, interventions should incorporate the following: (1) psycho-education, (2) practical adjustments at work to cope with cancer-related cognitive problems and fatigue, and they should focus on (3) acceptance and (4) communication about cancer-related cognitive problems at work. Furthermore, it is essential that professionals (both employers and (occupational) HCP) gain sufficient knowledge about cancer-related cognitive problems at work. Ensuring professional education regarding cancer-related cognitive problems, within both the healthcare and occupational health system, is of utmost importance. In addition, efforts are needed to improve coordination of care for cancer-related cognitive problems. Patient navigators (e.g., oncology nurses) may play a vital role in providing the link between cancer survivors, confronted with cancer-related cognitive problems at work, and (occupational) HCP. For example, Berezowska et al. (2019) [33] found that professional patient navigation is perceived as valuable by both cancer patients and HCP, and that patients apply the provided advice from their navigator in daily life activities, amongst which work. Future studies on mechanisms underlying cancer-related cognitive problems are important, as further knowledge about these mechanisms will help to develop cognitive rehabilitations interventions for cancer-related cognitive problems. Next, since the participants in this study were generally highly educated, non-manual workers, effort should be undertaken to involve the more vulnerable employees in qualitative studies like the current one. This is needed to be able to develop comprehensive interventions for not only the more affluent but also the more deprived persons in our society.

Finally, cancer-related cognitive problems are a major concern amongst working cancer survivors, their employers, but (occupational) HCP as well. The number of working cancer survivors dealing with cancer-related cognitive problems will continue to grow in the future in light of the rapid advances in cancer treatment that are leading to improved survival and consequent increased RTW opportunities. Therefore, there is an urgent need to support their employability, both from a cancer survivors, professional, and societal perspective.

### Electronic supplementary material

ESM 1 (DOCX 21 kb)
