# Supplementary material for: Cancer-related cognitive problems at work: experiences of survivors and professionals
Source: J Cancer Surviv. 2019 Nov 25;14(2):168–78. doi: 10.1007/s11764-019-00830-5 (PMC7182611; doi:10.1007/s11764-019-00830-5)
Supplement: Supplementary file 1 — (DOCX 21 kb) [file 11764_2019_830_MOESM1_ESM.docx]

**Supplementary file**

**Cancer-related cognitive problems at work: experiences of survivors and professionals**

*Journal of Cancer Survivorship*

Kete M. Klaver, MSc ^1,2^, Saskia F.A. Duijts, PhD ^2,3^, Ellen G. Engelhardt, PhD ^1,4^, Chantal A.V. Geusgens PhD ^5^, Maureen J.B. Aarts, PhD ^6^, Rudolf W.H.M. Ponds, PhD ^7^, Allard J. van der Beek, PhD ^2^, Sanne B. Schagen, PhD ^1,8^

^1^Division of Psychosocial Research and Epidemiology, Netherlands Cancer Institute, Amsterdam, The Netherlands, ^2^Department of Public and Occupational Health, Amsterdam Public Health research institute, Amsterdam UMC, Vrije Universiteit Amsterdam, Amsterdam, The Netherlands, ^3^Netherlands Comprehensive Cancer Organisation (IKNL), Utrecht, The Netherlands, ^4^Division of Molecular Pathology, Netherlands Cancer Institute, Amsterdam, The Netherlands, ^5^Department of Clinical and Medical Psychology, Zuyderland MC, Sittard, The Netherlands, ^6^Department of Medical Oncology, Maastricht University MC, Maastricht, The Netherlands, ^7^Department of Medical Psychology/School of Mental Health and Neurosciences (MHeNS), Maastricht University MC, Maastricht, The Netherlands, ^8^Brain and Cognition group, University of Amsterdam, Amsterdam, The Netherlands.

*Corresponding author*

Kete M. Klaver, MSc

Netherlands Cancer Institute

Division of Psychosocial Research and Epidemiology

Plesmanlaan 121, 1066 CX Amsterdam

E: k.klaver@nki.nl

T: +31 (0)20 512 6053

**Supplementary table 1:** Focus groups questions

| **Cancer survivors focus group questions** |
| --- |
| Cancer-related cognitive problems at work |
| - *Can you describe the cognitive problems you experience at work?* |
| - *What impact do these cognitive problems have at work?* |
| Coping with cancer-related cognitive problems at work |
| - *What did you do to cope with cognitive problems at work? How did that go?* |
| - *Are there people who helped you to cope with cognitive problems at work?* |
| Disclosure |
| - *Did you disclose your cognitive problems (in general and at work)? How did that go?* |
| - *What ease or strains did you experience in disclosing your cognitive problems at work?* |
| Treatment for cancer-related cognitive problems at work |
| - *Are you interested in treatment for cognitive problems at work?* |
| - *What topics should be incorporated in such a treatment?* |
| - *In what way would you like to be informed about treatment for cancer-related cognitive problems?* |
| **Professionals focus group questions** |
| Cancer survivors with cognitive problems at work |
| - *What are your experiences with cancer survivors with cognitive problems at work?* |
| - *What impact do cancer-related cognitive problems have at work?* |
| Support for cancer survivors with cognitive problems |
| - *What do you do to support cancer survivors with cognitive problems at work?* |
| - *What goes well and what has room for improvement?* |
| Disclosure |
| - *Do cancer survivors disclose their cognitive problems (in general and at work specifically)?* |
| - *How important is it to disclose cancer-related cognitive problems at work?* |
| Treatment or cancer-related cognitive problems at work |
| - *What topics should be incorporated in a treatment for cancer-related cognitive problems?* |
| - *If there would be evidence based treatment options for cancer-related cognitive problems at work, would you refer your employee/ client?* |
| - *In what way should cancer survivors be informed about treatment for cognitive problems at work?* |
|  |

**Supplementary table 2:** Characteristics of cancer survivors participating in the focus groups

| p ͣ | Age | Educational level ^b^ | Treatment ^c^ | Job sector | Work hours per week |
| --- | --- | --- | --- | --- | --- |
| p1 | 65 | Low | S; RT; C | Transport | 40 |
| p2 | 60 | High | S; RT; C; E | Health care | 20 |
| p3 | 61 | High | S | Financial | 40 |
| p4 | 68 | High | S; RT; C | Financial, industry and construction | 30 |
| p5 | 32 | High | S; RT; C | Education | 40 |
| p6 | 44 | High | S; RT; C; E | Education | 40 |
| p7 | 62 | High | S; C; E; I | Health care | 16 |
| p8 | 62 | High | S; RT; E; O | Health care | 28 |
| p9 | 39 | Moderate | S; C; E | Trade | 28 |
| p10 | 41 | High | C | Public services | 12 |
| p11 | 46 | High | S; RT; C; E | Culture, recreation | 32 |
| p12 | 42 | High | RT; C | Health care | 27 |
| p13 | 46 | Moderate | S; C | Energy and mining | 32 |
| p14 | 44 | High | S; RT; C | Culture | 40 |
| p15 | 48 | High | S; RT; C | Transport | 40 |
| p16 | 49 | High | RT;C; O | Trade | 40 |
| p17 | 70 | High | RT; I | Culture | 40 |
| p18 | 31 | High | S; RT; C; I | Transport | 28 |
| p19 | 44 | High | S; I | Financial | 32 |
| p20 | 36 | High | S | Financial and construction | 36 |
| p21 | 50 | Moderate | S | Health care | 28 |
| p22 | 41 | High | S; O | Education | 16 |
| p23 | 57 | High | S | Industry | 40 |

ͣ Participant number

ᵇ Low: Vocational education; Moderate: Senior secondary vocational education; High: Higher professional

^c^ Multiple answers were possible; S: surgery; RT: radiotherapy; C: chemotherapy; E: endocrine therapy; I: immunotherapy; O: other
